# Joint Raman spectroscopic and quantum chemical analysis of the vibrational features of Cs_2_RuO_4_

**DOI:** 10.1002/jrs.4705

**Published:** 2015-04-28

**Authors:** M Naji, F Di Lemma, A Kovács, O Beneš, D Manara, J-Y Colle, G Pagliosa, P Raison, R J M Konings

**Affiliations:** European Commission, Joint Research Centre (JRC), Institute for Transuranium Elements (ITU)Postfach 2340, 76125, Karlsruhe, Germany

**Keywords:** fission products, nuclear reactor materials, phase transition, quantum chemical analysis, ruthenium compounds

## Abstract

The Raman spectroscopic characterization of the orthorhombic phase of Cs_2_RuO_4_ was carried out by means of group theory and quantum chemical analysis. Multiple models based on ruthenate (VI+) tetrahedra were tested, and characterization of all the active Raman modes was achieved. A comparison of Raman spectra of Cs_2_RuO_4_, Cs_2_MoO_4_, and Cs_2_WO_4_ was also performed. Raman laser heating induced a phase transition from an ordered to a disordered structure. The temperature-phase transition was calculated from the anti-Stokes/Stokes ratio and compared with the ones measured at macroscopic scale. The phase transition is connected with tilting and/or rotations of RuO_4_ tetrahedra, which lead to a disorder at the RuO_4_ sites. © 2015 The Authors. *Journal of Raman Spectroscopy* published by John Wiley & Sons Ltd.

## Introduction

Much attention has been devoted to the study of the behavior of the ruthenium fission product in nuclear reactor accidents.^1^–^9^

This is because of several factors: its high specific activity,^6^ high radio-toxicity for its isotopes ^106^Ru (half-life = 369 days) and ^103^Ru (half-life = 39.3 days),^7^–^9^ and also the increase of its quantity with the fuel burn-up (Ru yield is higher for ^239^Pu than ^235^U).^3^

In the case of nuclear severe accidents, mainly gaseous and volatile fission products are released from the damaged fuel, while transition metals such as ruthenium present in the irradiated fuel would not be released to any significant extent. However, in highly oxidizing atmospheres, especially under steam air conditions, metallic ruthenium can be oxidized to volatile RuO_3_ and RuO_4_ at moderately high temperature and almost completely released from the damaged fuel.^10^ According to the Ellingham diagram, the presence of volatile ruthenium oxides occurs only if the fuel elements (uranium and plutonium) have previously been oxidized.^11^ Important conclusions concerning the ruthenium release can be drawn also from the Chernobyl accident. Indeed, the total release of ^103^Ru was higher than that of ^137^Cs, corresponding to about 2.9% of the Ru inventory at the start of the accident.^12^,^13^ Thus, confirming the possibility of the release of ruthenium outside of the fuel matrix.

When it is released out of the fuel matrix, ruthenium can be found in various physico-chemical forms, either in simple oxide like RuO_3_ and RuO_4_ or in the form of mixed compounds with alkali metals like Cs, e.g. as Cs_2_RuO_4_, and alkali earth metals (Ba and Sr) present in large quantity as fission products.^3^ Moreover, possible formation of Cs_2_RuO_4_ could increase the volatility of Ru during an accident.^1^ The necessity and importance of studying the Cs-Ru-O system stand out first because of the radiological hazards posed by the volatile nature of its oxides and second to make best estimate assessment in terms of nuclear safety issues.

In terms of thermodynamic features, the system Cs-Ru-O was only rudimentary investigated,^14^–^17^ no reliable phase diagram has been reported so far in the literature, most probably because of the unstable character of ruthenium compounds.

In terms of structure, Cs_2_RuO_4_ belongs to compounds with the general formula A_2_BX_4_.^18^ These are known to crystallize in different types of structures like olivine, spinel, and other incommensurate phases.^19^ The rich sequence of structural phases makes the study of such compound challenging and calls for a fundamental understanding of the orientational ordering of BX_4_ tetrahedra responsible for structure variations.

In other frames, Raman spectroscopy has been known to provide useful structural information in various hostile environments like in hot cells.^20^,^21^ Thus, vibrational studies of such materials are of great importance in identifying fission products and their corresponding phases in the fuel. Consequently, in order to address a part of these issues, we propose to investigate the structure of Cs_2_RuO_4_ at room and high temperatures. We provide a complete vibrational study of this compound by means of Raman spectroscopy. We succeeded to combine group theory analysis (GTA) and quantum chemical calculation to characterize all of the observed Raman bands. We revealed a phase transition, induced locally by Raman laser heating experiment.

## Experimental

### Material synthesis

The polycrystalline sample Cs_2_RuO_4_ was prepared by solid-state reaction of Cs_2_O and RuO_2_, following the same procedure described by Cordfunke *et al*.^15^ Cs_2_O was obtained from the decomposition of Cs_2_CO_3_, performed in a furnace at 925 K overnight in a constant flow of purified O_2_ gas. The material was contained in an iridium–platinum boat and heated with a ramp of 10 K/min. Its purity was checked by X-ray diffraction (XRD). Stoichiometric proportions of Cs_2_O (as-prepared) and RuO_2_ (as-received from Sigma-Aldrich, 99.9%) powders were intimately mixed and grounded in an agate mortar and subsequently heated in a flow of purified oxygen. The temperature was increased in a stepwise from room temperature to 1075 K. Below 700 K, the sample was heated in a silver boat, and above that temperature (up to 1075 K), a gold boat was necessary. The heat treatment sequence is reported in Table S1 (Supporting Information). The purity of the final compound was checked by room temperature XRD, and the pattern shows only the reflection peaks corresponding to Cs_2_RuO_4_. No secondary phases could be detected by XRD and the material appeared to be of a satisfactory purity (Bragg peaks of unidentified spurious phases were <1% in intensity). As the compound is highly hygroscopic, it was handled all the time in a glove box under inert atmosphere.

### Instrumental methods

#### X-ray diffraction

The crystal structures of Cs_2_RuO_4_ reported by Fischer and Hoppe^14^ was revisited at room temperature by XRD on polycrystalline material. The data collection was performed on Bruker D8-Advance X-ray diffractometer mounted in the Bragg–Brentano configuration with a curved germanium monochromator (111), using CuK_α1_ = 1.5406 Å radiation and a LinxEye position sensitive detector. The data were collected between 7° and 130° with steps of 0.0092° (*2θ*) with an integration time of 26 : 40 h. The equipment is placed in a glove box under inert atmosphere. The powder was mixed with an Epoxy resin to avoid reaction with humidity during the measurement. The structural analysis was performed by the Rietveld method with the Fullprof2k suite.^22^ The refined structural parameters for Cs_2_RuO_4_ are given in Table1 and show a good agreement with those reported by Fischer and Hoppe.^14^ Figure S1 (Supporting Information) illustrates the agreement between the observed and the calculated diffraction profiles for Cs_2_RuO_4_. The atomic positions in the Cs_2_RuO_4_ are given in Table S2 (Supporting Information). Full crystal structure analysis (bond distances/angles, etc.) of Cs_2_RuO_4_ reveals that RuO_4_ tertahedra are highly distorted as compared with the crystal structure published by Fischer and Hoppe.^14^ This can reflect a lack of accuracy on the oxygen positions. Therefore, for our Raman quantum analysis calculations, we have used the crystal data published in literature.^14^

**Table 1:** Unit cell parameters and results of the Rietveld refinement for Cs_2_RuO_4_ (crystalline powder) compared with those from single crystal^14^

Formula unit	Cs_2_RuO_4_	
	This work	Fischer and Hoppe^14^
a	8.506(1) Å	8.512(1) Å
b	6.472(1) Å	6.475(1) Å
c	11.460(1) Å	11.458(2) Å
V	630.9 Å^3^	631.5 Å^3^
Space group	Pnma (*N*°= 62)	Pnma (*N*°= 62)
Z	4	4
Refined parameters	67	
R_wp_	4.53%	
R_P_	3.11%	
R_B_	7.50%	
R_exp_	2.13%	
GOF	2.11	

R_P_ = Σ[y_i_(obs) − y_i_(calc)]/Σy_i_(obs); R_wp_ = {Σw_i_[y_i_(obs) − y_i_(calc)]^2^/Σy_i_(obs)}^1/2^.

R_B_ = Σ[I_hkl_(obs) − I_hkl_(calc)]/ΣI_hkl_(obs); GOF = R_wp_/R_exp_.

GOF, Goodness of the Fit.

### Raman spectroscopy

Raman micro-spectroscopy measurements were carried out with a Horiba Jobin–Yvon T64000 spectrometer used in the single (for Stokes lines acquisition) and in the triple additive spectrograph configuration (for anti-Stokes/Stokes measurements). Raman backscattering was excited with an Ar^+^ laser working at 514.5 nm (2.41 eV). The crystalline powder was loaded inside an argon glove box into a Plexiglas sample holder with a quartz window and was subsequently sealed. The laser was then focused onto the sample using an objective of ×50. The power at the surface of the sample was measured by Coherent power meter placed at the sample position. The Raman spectrometer was calibrated using a Si single crystal, and the correct shift was maintained for all samples. A calibrated white light source combined with a fiber optic was used to correct Raman spectra for instrumental response. The recorded ‘response’ spectrum was compared with a ‘reference’ spectrum having a known output of intensity *versus* wavelength. The correction function *f*(*λ*) is calculated as follows:

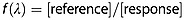
1

Each spectrum acquired is multiplied by the correction factor to yield the corrected spectrum.

### Computational details

The density functional theory calculations have been performed by means of the Gaussian09 code^23^ using the B3LYP exchange-correlation functional.^24^,^25^ The small-core relativistic pseudopotentials of the Cologne–Stuttgart group were used for the heavy atoms: that of Ru and Mo contained 28 electrons in the core ([Ar] 3d^10^, ECP28MDF),^26^ that of Cs 46 ([Kr] 4d^10^, ECP46MDF),^27^ and that of W 60 electrons ([Kr] 4d^10^4f^14^, ECP60MDF).^28^ Basis sets of quadruple-zeta quality were applied for the 4s, 4p, 4d, and 5s electrons of Ru and Mo,^26^ for the 5s, 5p, and 6s electrons of Cs,^27^ and for the 5s, 5p, 5d, and 6s electrons of W.^28^ For oxygen, the correlation-consistent cc-pVQZ basis set^29^ was used. Both singlet and triplet spin multiplicities were considered in the model structures containing Ru. The geometry optimizations were performed without any geometry constraint. The harmonic vibrational wavenumbers together with Raman activities were calculated for these optimized geometries. In order to be consistent with the experimental Raman spectra, the calculated Raman activities were corrected for the wavelength of the excitation laser line (514.5 nm, Ar).^30^,^31^

## Results and discussion

**Figure 1:**
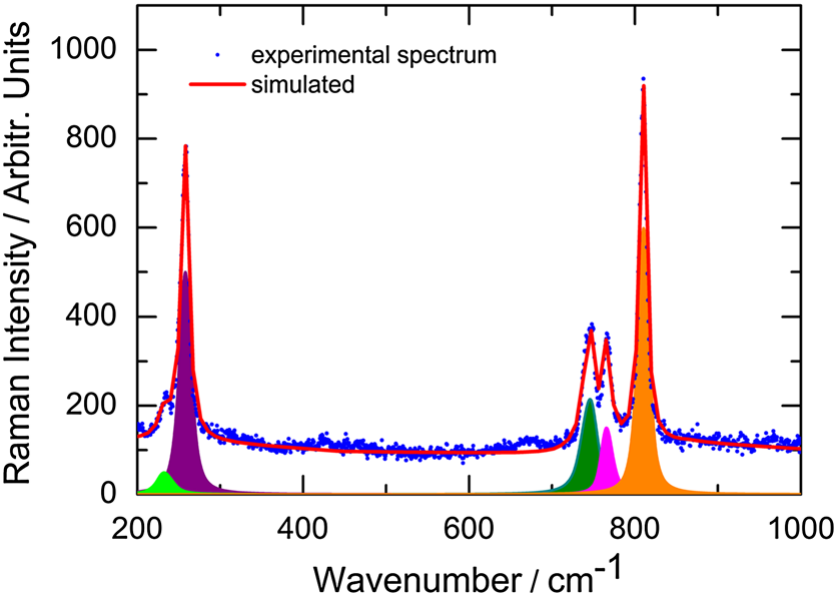
Room temperature Raman spectrum of Cs_2_RuO_4_ (full circles) simulated with a sum of Lorentz bands (solid line). Power at the surface of the sample was 2 mW.

The Raman band characterization of Cs_2_RuO_4_ was performed by a coupling of symmetry selection rules as predicted by group theory and quantum chemical analysis. Figure1 shows a representative Raman spectrum of Cs_2_RuO_4_ in the 200–1000 cm^−1^ wavenumber range, measured at room temperature for an excitation line of 514.5 nm and a power of 2 mW at the sample surface. To extract the damping coefficients and wavenumbers of the characteristic modes, the spectrum was fitted to a sum of Lorentzian lines (Fig.1). Table2 lists all the Raman wavenumber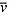 and the damping coefficient *Γ* of the observed bands. All bands exhibit a rather narrow width and are located below 1000 cm^−1^, which is the usual case for these kind of A_2_XO_4_ (A = Na, K, Rb, Cs and X = Mo, W) compounds.^32^–^37^ Moreover, except for a change of the background shape when changing the excitation energies (from 2.41 to 1.92 eV), we did not observe any significant changes either in the intensity or in the wavenumber of these modes, confirming the non-resonant character of the observed modes.

**Table 2:** Comparison of experimental Raman spectrum of Cs_2_RuO_4_ with the calculated ones of the models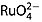, Cs_2_RuO_4_, and
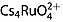

Experimental Raman		Calculated			Character
		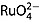	Cs_2_RuO_4_ (*D*_2*d*_)	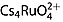 (*D*_2*d*_)	
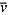 (cm^−1^)	Γ (cm^−1^)	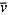 (cm^−1^), irreducible representations			
95 w (*A_g_*)			97 (*A*_1_)	72 (*A*_1_)	RuO_2_ bend + CsO asym stretch
105 w (*B*_3*g*_)			148 (*E*)	88 (*E*)	CsO sym stretch
245 w (*A_g_*)	16.65	242 (*T*_2_)	325 (*A*_1_)	248 (*A*_1_)	RuO_2_ sym bend
270 s (*B*_1*g*_)	10.88	249 (*E*)	233 (*B*_1_)	303 (*B*_1_)	RuO_2_ twist
770 m (*B*_2*g*_)	16.6		788 (*B*_2_)	767 (*B*_2_)	RuO asym strech
780 m (*B*_3*g*_)	9.85	747 (*T*_2_)	767 (*E*)	780 (*E*)	RuO asym strech
810 w (*A_g_*)	9.68	808 (*A*_1_)	835 (*A*_1_)	837 (*A*_1_)	RuO sym strech

### Group theory analysis

The room temperature of Cs_2_RuO_4_ crystal structure belongs to space group Pnma with one ruthenium, two caesium, and three oxygen independent atoms, and it has four formula units per unit cell.^14^

Applying the GTA, the fundamental modes at the *Γ* point (*k* = 0) are distributed in terms of the irreducible representations of the factor group *D*_2*h*_ as follows:

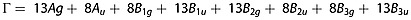
where *B*_1*u*_, *B*_2*u*_, and *B*_3*u*_ are acoustic modes. The vibrations belonging to *A_g_*, *B*_1*g*,_ *B*_2*g*_, and *B*_3*g*_ irreducible representations are Raman active modes.

**Figure 2:**
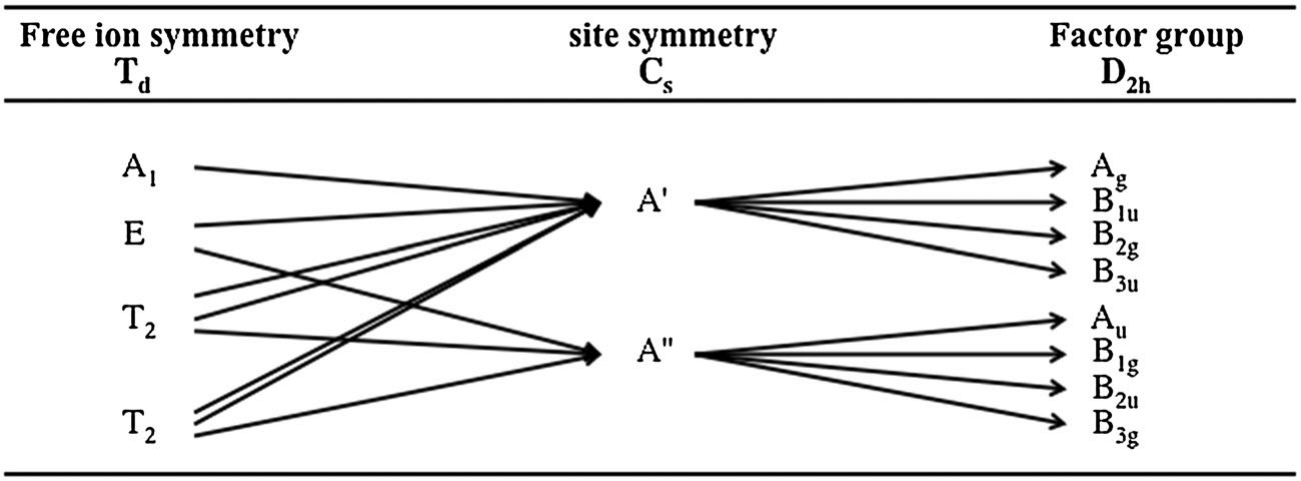
Correlation diagram for RuO_4_^2−^ in the orthorhombic structure. Correlation between *T_d_* point group, *C_s_* site symmetry, and *D*_2*h*_ factor group.

Because the polarized Raman analysis is not possible in this case (crystalline powder), in order to make a complete characterization of the Raman modes of Cs_2_RuO_4_ in its orthorhombic phase, we took benefit of two observations: First, the Cs_2_RuO_4_ spectrum is made of two main envelopes – one asymmetric at low wavenumber and a triplet at high wavenumber. The latter is very likely the sum of bands split from a vibrational mode of higher symmetry. Second, the Cs_2_RuO_4_ crystalline structure is made of isolated ruthenate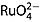 species. Hence, some correlations should exist between their vibrational modes. Therefore, we used the correlation diagram between the ‘free ion ➔ site group ➔ factor group’ to predict the allowed fundamental modes and their consequent splitting (crystal field effect). The correlation diagram is presented in Fig.2

The free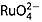 ion in *T_d_* symmetry exhibits four vibrational Raman active modes *A*_1_, *E*, and two *T*_2_. The atomic vector displacement analysis performed through the analysis of the symmetry adapted modes for a given orbit shows that two modes are predominately of stretching type *ν*_1_(*A*_1_) and *ν*_3_(*T*_2_), and the rests are bending *ν*_2_(*E*) and *ν*_4_(*T*_2_). Raman tensors related to these irreducible representations predict that all the modes except for *A*_1_ have non-zero polarizability coefficients in the off-diagonal matrix. Therefore, only the *A*_1_ mode is polarized. In the Cs_2_RuO_4_ crystal, the site symmetry is lower than *T_d_*, and the site group analysis allows us to predict the consequent splitting of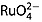 ion modes. The doubly and triply degenerate modes (*E* and *T*_2_) are smoothed out, resulting in the emergence of two bands for *ν*_2_ (*A*′ + *A*″) and three bands for *ν*_3_ and *ν*_4_ (2*A*′ + *A*″). Moreover, the crystal field effect leads to the splitting into eight non-degenerate modes: four for *A*′ and four for *A*″ symmetries (Fig.2). As aforementioned, only vibrations belonging to *A_g_*, *B*_1*g*_, *B*_2*g*_, and *B_3g_* are Raman active.

Heretofore, we have used the GTA analysis to predict all the Raman active modes for Cs_2_RuO_4_, and we correlated them to those of the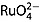 free ion. Now, to assist the characterization of the Raman spectrum shown in Fig.1, we have performed quantum chemical calculations.

### Quantum chemical analysis

Properly chosen small model structures have often been successfully applied in the literature for characterization of the main vibrational properties in crystalline and non-crystalline systems.^38^–^41^ Such models work well in cases when the main moiety of the compound interacts only weakly with the surroundings. The weak interactions of the RuO_4_^2−^ moiety with Cs in crystalline Cs_2_RuO_4_ satisfy this requirement.

**Figure 3:**
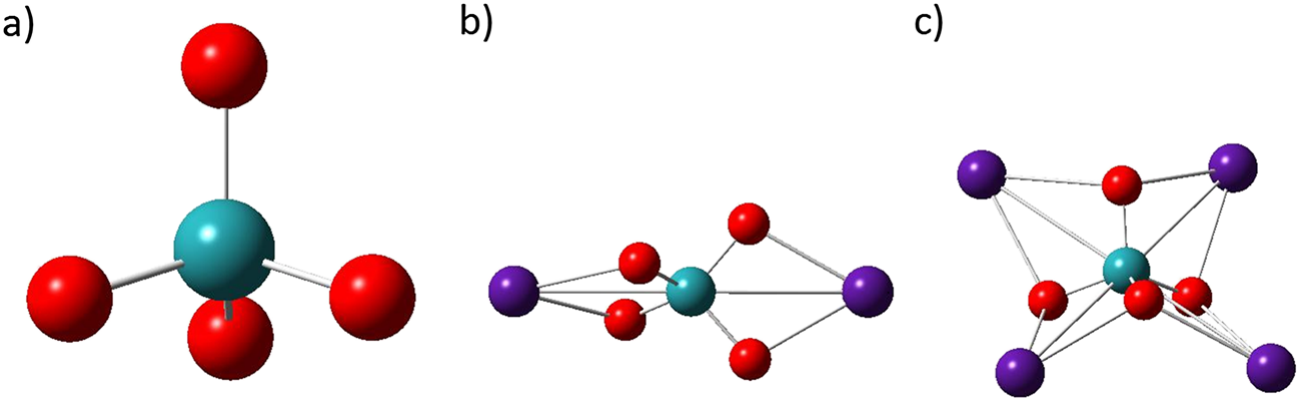
The model structures of (a) RuO_4_^2−^, (b) Cs_2_RuO_4_, and (c) Cs_4_RuO_4_^2+^.

**Figure 4:**
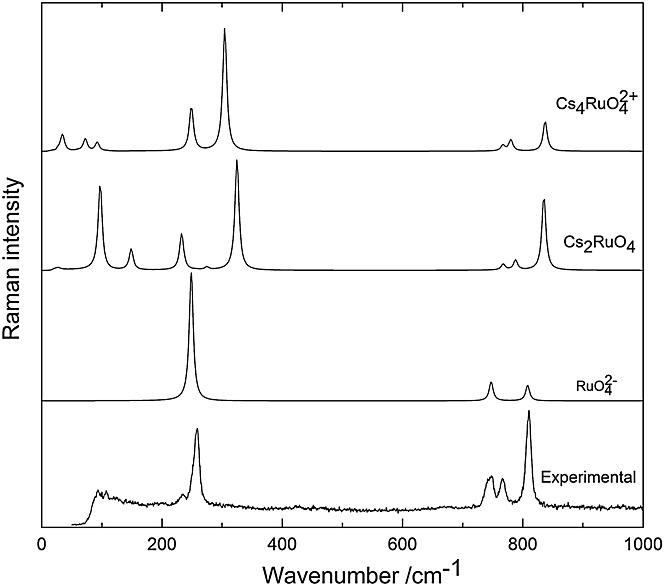
Comparison of the experimental Raman spectrum of solid Cs_2_RuO_4_ (bottom) with the calculated ones of the models RuO_4_^2−^, Cs_2_RuO_4_, and Cs_4_RuO_4_^2+^.

We probed three model structures (Fig.3) for description of the vibrational spectra of solid Cs_2_RuO_4_. In the crystal, the RuO_4_ moieties are distinct units: each Ru is four coordinated and the four oxygens surround the metal in a distorted tetrahedral arrangement. Our first (simplest) model was the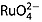 ion. From the possible two spin multiplicities (singlet and triplet), the triplet one is more stable (by 70 kJ/mol); hence, we considered only the data for that state. The Ru-O bond distances of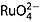 are in good agreement with the experimental ones (cf. Table3). Similarly, the calculated Raman spectrum describes well the main features of the experimental Raman spectra (Fig.4). These are the symmetric and one of the asymmetric RuO stretching modes at around 800 cm^−1^ and the twisting deformation mode at around 270 cm^−1^. The wavenumbers are in remarkably good agreement (cf. Table2), while the calculated Raman intensities are too strong in the low-wavenumber part of the spectrum.

**Table 3:** Comparison of selected geometrical parameters (Å, deg.) of Cs_2_RuO_4_ with the calculated ones of the models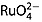, Cs_2_RuO_4_, and
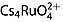

Parameter	X-ray (Fischer and Hoppe^14^)	Calculated		
	Cs_2_RuO_4_	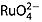	Cs_2_RuO_4_	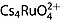
Ru-O	1.752–1.774	1.785	1.777	1.777
Cs-O	3.037–3.126	—	2.764	3.026
O-Ru-O	107.4–111.9	109.5	102.9	118/105.4
Ru-O-Cs	106.1–160.8	—	98.4	99.5

The RuO_4_ moieties are connected by Cs atoms in the crystal, and these Ru-O-Cs interactions (resulting also in the distortion of tetrahedral RuO_4_) modify somewhat the vibrations of the RuO_4_ moiety with respect to the simple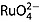 model. In addition, CsO vibrations appear in the spectra too. The interactions of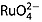 with Cs atoms were assessed by two model structures: Cs_2_RuO_4_ and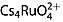. The second model with two Cs atoms around each oxygen resembles more the surrounding of oxygens in the crystal. Again two spin multiplicities (singlet and triplet) were considered, and the triplets were found to be more stable by 52 and 34 kJ/mol for Cs_2_RuO_4_ and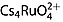, respectively. These triplet data are presented and discussed in the following.

From the two models, the geometrical parameters of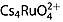 agree well with the crystal structure data (cf. Table3). The computed Ru-O bond distance is marginally larger, while the Cs-O bond distance is marginally shorter than those determined for the crystal. Because of the inherent Cs-O interactions in Cs_2_RuO_4_ as compared with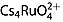 and the solid state, the Cs-O bonds are considerably (by ˜0.3 Å) shorter in Cs_2_RuO_4_. This results in considerably stronger Cs-O interactions in the latter model but does not affect notably the Ru-O interactions, because the Ru-O bond distance is exactly the same as in the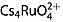 model. Therefore, on the basis of the geometrical properties, both models are expected to describe well the vibrations of the RuO_4_ moiety, while Cs_2_RuO_4_ may fail for fundamentals containing significant CsO contribution.

The calculated vibrational spectra of the two models are compared with the experimental one in Fig.4. Both models describe well the RuO stretching range of the Raman spectrum with the one intense and two weaker bands in the range of 700–900 cm^−1^. Also the intense RuO_2_ twist around 300 cm^−1^ can be well recognized in the spectra of the two models. Hence, the calculations support the attributions of the RuO stretching and twisting vibrations based on the RuO_4_^2−^ model. The weak fundamentals at 325 and 248 cm^−1^ (having the same symmetry and character) in the calculated spectra of Cs_2_RuO_4_ and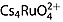, respectively, may be associated with the weak feature at 245 cm^−1^ in the experimental spectrum. According to the calculations, it may correspond to RuO_2_ bending. We note that the wavenumber of this vibration in Cs_2_RuO_4_ is higher by ˜80 cm^−1^ because of the more strained O-Cs-O arrangement in this model structure.

In general, the calculated Raman spectrum (both the wavenumbers and intensities) of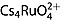 is in somewhat better agreement with the experimental spectrum than the calculated one of Cs_2_RuO_4_. The latter shows two bands below 200 cm^−1^ with considerable intensity, which cannot be recognized in the experimental spectrum. Obviously, this is the consequence of the different CsO interactions in this model. According to the calculations, the CsO stretching modes can be expected between 75 and 300 cm^−1^ mostly mixed with RuO_4_ deformation, while the CsO deformation vibrations mixed with RuO_4_ deformation are probably below 100 cm^−1^. Two very weak bands can be recognized around 100 cm^−1^ in the Raman spectrum of the solid. The computed data and the proposed characterization of the experimental Raman bands are compiled in Table2. We note the opposite order in the wavenumbers of the *A*_1_/*B*_1_ deformation and *B*_2_/*E* asymmetric stretching vibrations of the Cs_2_RuO_4_ and Cs_4_RuO_4_^2+^ models. This is due to the different arrangements of Cs around the RuO_4_^2−^ moiety in the two model structures.

### Comparison with ruthenate and other tetraoxy-species

As stated earlier, vibrational data on ruthenium tetraoxide and related compounds are lacking from literature. Therefore, we compared our results with those found for liquid ruthenium tetroxide RuO_4_ and with the one of crystalline K_2_RuO_4_.^42^,^43^ The Raman spectrum of liquid ruthenium tetroxide exhibits four bands: polarized intense band at 882 cm^−1^ attributed to *ν*_1_(*A*_1_), a depolarized band at 323 cm^−1^ assigned to *ν*_2_(*E*), and two additional bands at 914 and 334 cm^−1^ to be *ν*_3_(*T*_2_) and *ν*_4_(*T*_2_), respectively,^42^,^43^ while for the isostructural K_2_RuO_4_, the Raman spectrum presents Raman bands related to *ν*_1_, *ν*_2_, *ν*_3_, and *ν*_4_.^43^ Except for a slight shift in the wavenumbers, our spectrum agrees well with literature. Unfortunately, other oxo-ruthenate-related compounds such as Na_2_RuO_4_, cannot be taken into consideration for a comparison purpose because they do not have the same tetrahedral structural units.^44^,^45^ In these compounds, ruthenium atoms were found to form trigonal bipyramids.^44^,^45^ Consequently, we have compared the vibrational properties of Cs_2_RuO_4_ with its molybdate and tungstate analogues. Raman spectra of Cs_2_MoO_4_ and Cs_2_WO_4_ were recorded at room temperature under the same conditions described earlier.

**Figure 5:**
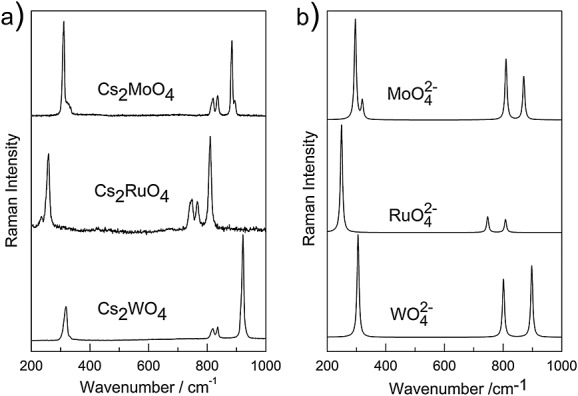
(a) Experimental Raman spectra of Cs_2_WO_4_, Cs_2_RuO_4_, and Cs_2_MoO_4_ at room temperature. Power at the surface is 2 mW. (b) Computed Raman spectra of the WO_4_^2−^, RuO_4_^2−^, and MoO_4_^2−^moieties.

In contrast to cubic systems of M_2_XO_4_ (M = Na, K) and (X = Mo, W),^33^,^35^ the analogues caesium compounds crystallize in an orthorhombic symmetry. Within the instrumental uncertainty, the phonon wavenumbers of Cs_2_MoO_4_ and Cs_2_WO_4_ shown in Fig.5(a) agree well with those reported in literature.^46^ We note that for Wallez *et al*.,^46^ the band centered at 881 cm^−1^ for Cs_2_MoO_4_ was reproduced with only one mode, whereas in our spectrum, we observe a small shoulder at 893 cm^−1^ too. A similar band was observed in the high-temperature orthorhombic phase of Na_2_MoO_4_ as well as in its room temperature hydrated one Na_2_MoO_4_.xH_2_O (orthorhombic phase), that is due to the high distortion of the tetrahedral molecular symmetry of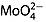.^34^ Regarding Cs_2_WO_4_, its phonon wavenumbers fit well with the ones reported for Rb_2_WO_4_.^36^ The computed Raman spectra of the WO_4_^2−^, RuO_4_^2−^, and MoO_4_^2−^ species show the same characteristic differences as the experimental ones (Fig.5(b)).

Comparing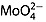 with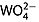, the fundamental modes of the latter moiety are generally blue shifted. The larger stretching wavenumber of the WO_4_ moiety as compared with MoO_4_ is attributed to the larger relativistic effects in W, which result in shorter bond distance and larger stretching force constant for WO_4_. This feature characteristic for elements in the fifth and sixth rows of the periodic table is well documented.^47^,^48^ As the shorter bond distances mean larger electron densities within the bonds, their repulsion can result in larger bending force constants too. Hence, the blue shift of the fundamentals of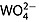.

As can be seen in Fig.5, all the bands of Cs_2_RuO_4_ are red shifted with respect to Cs_2_MoO_4_. This is in agreement with the larger mass of Ru with respect to Mo (being in the same row of the periodic table). The magnitude of the shift, however, may suggest an additional origin. In Cs_2_XO_4_ (Ru, Mo, and W), the electronic configuration of Ru(VI+) is [Kr]4d^2^, whereas Mo(VI+) and W(VI+) exhibit both *d*^0^ configuration in their valence shell. The electronic structure of the central atom directs towards tetra coordination. While for Cs_2_XO_4_ (X = Mo, W, Ru), the coordination number of the central atom is the same, the ligand field is different. In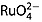, a ligand field stabilization for *d*^2^ occurs being different from those for *d*^0^ configuration. This may induce an effect resulting in a loosening of the force constants and consequently in red shifts.

### Laser heating-induced phase transition

**Figure 6:**
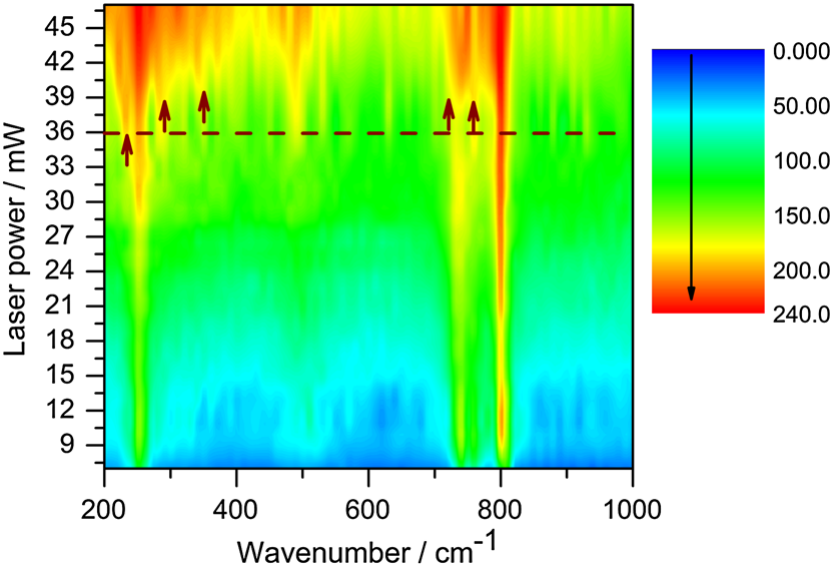
Three-dimensional plot showing the dependence on laser heating for the Raman spectra of Cs_2_RuO_4_ heated with 514 nm (Ar^+^) laser. The *x*-axis represents the wavenumbers, *y*-axis shows the laser power measured at the sample surface, and *z*-axis (change of colors) represents the variation of Raman intensity. Intensity increases from blue to red. Arrows indicate appearance of Raman modes above 36 mW (shown with dashed line).

Laser Raman spectroscopy represents a powerful tool for the *in situ* investigation of structural changes. A large number of laser-induced phase transitions and oxidation studies using this technique have been conducted on a wide range of materials. In this experiment, we irradiated the sample surface with different powers and simultaneously collect the Raman spectra. Figure6 represents a three-dimensional plot of Raman spectra acquired at different laser powers. The *x*-axis corresponds to the wavenumber, the *y*-axis is the laser power measured at the sample surface, while the color change represents the change of the Raman intensity in increasing scale from blue to red. Individual Raman spectra are *x–z* cut. In this plot, each spectrum has been divided by its average value and multiplied by constant (color scale factor) to correct global intensity fluctuations, which might originate from defocusing, and possible laser intensity fluctuations. In this way, one can have a rapid and general view of the whole set of data acquired. Moreover, small variations are much easier to visualize, as they are too small to appear unambiguously on the simple comparison of two spectra, because of insufficient signal-to-noise ratio. This method has been applied in similar Raman studies on different materials where the spectral changes are not obvious.^49^,^50^ Upon increasing the laser power in the 2–36 mW power range, the Raman spectra remain qualitatively the same. The wavenumber of all peaks exhibits relatively weak power dependence, except for the band at 237 cm^−1^, which exhibits a weak bandwidth change due to the anharmonic effect introduced by the increase of temperature when the laser power is increased. The Raman spectra change drastically above 36 mW (dashed line in Fig.6). The changes in the Raman spectra are very impressive, i.e. some Raman bands split and new bands appear in the wavenumber range between 237–400 and 700–850 cm^−1^ (illustrated with arrows in Fig.6). Appearance of new modes reveal in most cases a reduction of the crystal symmetry, here most probably because of rotational/translational changes of RuO_4_ tetrahedra. Furthermore, appearance of these new modes in the Raman spectra is accompanied with an increase of the bandwidths of the observed bands indicating that some disorder is introduced in the new structure.

A phase transition for Cs_2_RuO_4_ was observed by differential scanning calorimetry (DSC) at 907 K by Ball *et al*.^51^ Moreover, some unpublished high-temperature phases can be found in the International Center for Diffraction Database for Cs_2_RuO_4_, these being related to a hexagonal and a tetragonal crystal structure. The former was obtained at 975 K by van Vlaanderen,^52^ while the latter one by Range at 423 K.^53^ Finally, a phase transition has been reported for the isostructural compound Cs_2_MoO_4_ by Wallez *et al*.^46^ Furthermore, high-temperature XRD measurement was performed to evaluate the phase transition of Cs_2_RuO_4_ (not shown here). At around 875 K, the appearance of new bands in the XRD pattern could be observed, this transition was completed at 975 K. The new pattern is similar to the one of van Vlaanderen.^52^

The scenario of the laser-induced phase transformation could be proposed as follows: from a thermal point of view, the large temperature gradient present in the spot heated by the laser will result in thermal stresses. These induce a gradient of lattice constants and enhance the degree of freedom of species. Thus, permit the rotation and also translation of RuO_4_ tetrahedra and Cs atoms, resulting in a more disordered system as indicated by the broadening of the Raman bands. Consequently, the system undergoes a phase transition similar to that reported for Cs_2_MoO_4_.^46^

**Figure 7:**
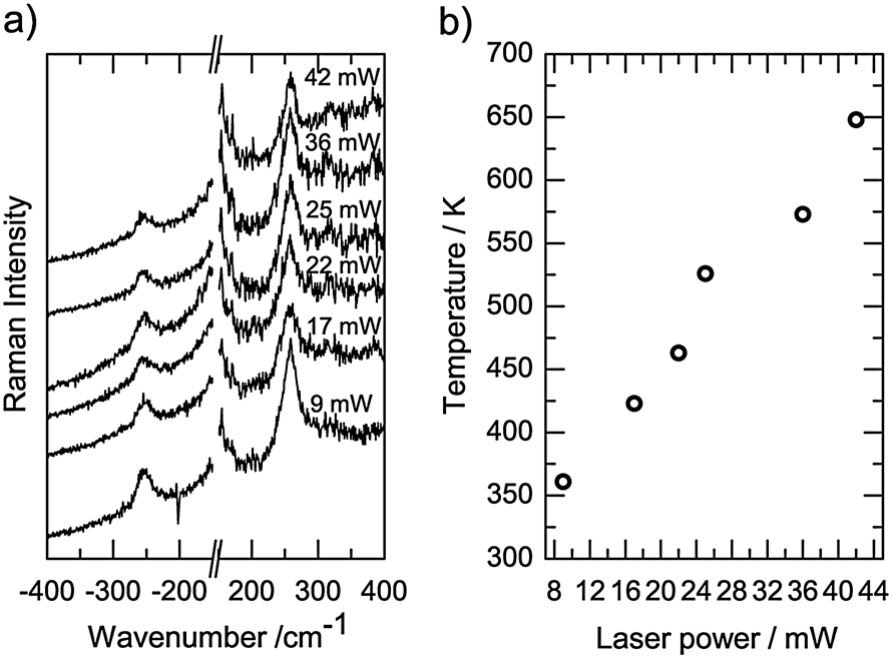
(a) Selected Raman spectra collected from Cs_2_RuO_4_ with a stepwise increase in laser power showing the Stokes and anti-Stokes low-wavenumber region (200–400)cm^−1^. (b) Correlation of the laser power with a local temperature of the sample evaluated with the method described in Eqn (2).

The thermal effect of a laser beam is the result of its absorption by the sample. It is evident that the absorptivity of Cs_2_RuO_4_ in the wavelength range of the laser is very high as shown by the red shifts of bands and also the increase in bandwidths. At laser power higher than 36 mW, the heating effect is directly observed by the charge-coupled device camera image of thermal radiation from the laser-heated spot. To estimate the temperature at the surface of the sample, different methods can be used. In this study, we have recorded the Stokes and anti-Stokes lines as a function of the laser power. Figure7(a) shows some selected Stokes and anti-Stokes Raman spectra of Cs_2_RuO_4_ obtained with a stepwise increase in laser power. As described in the experimental methods, the intensities were corrected for the spectral responses of the optical system and the charge-coupled device detector (quantum efficiency), and the sample was maintained in a sealed sample holder. Using the following equation (Eqn (2)) based on the Boltzmann distribution of modes between energy levels, one can give a good estimate of the temperature at the surface of the irradiated spot.

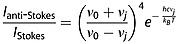
(2)where *ν*_0_ and *ν*_*j*_ are the wavenumbers of the excitation line and the Raman modes, respectively, *I*_anti - Stokes_ and *I*_Stokes_ are the intensities of the anti-Stokes and Stokes Raman peaks, *k_B_* is the Boltzmann constant, *c* is the speed of light, and *T* is the temperature. Intensities and wavenumbers of the *B*_1*g*_ mode at 270 cm^−1^ were used as input parameters. Figure7(b) shows the calculated correlations between the laser power and sample temperature.

The sample temperature calculated from the anti-Stokes/Stokes method gives rather satisfactory results, and we estimate a phase-transition temperature at around 571 K. The estimation of the accuracy seems to be difficult especially at high powers where the fluorescence level in the spectra becomes more dominant. Furthermore, as all the observed modes broaden at high temperature, the evaluation of their corresponding intensities can be also a source of uncertainty, mainly because of band overlapping (Fig.7(b)).

Prior to concluding this study, one has to comment the phase-transition temperatures obtained from Raman spectroscopy (571 K) and from DSC and XRD data (875–975 K). The temperature measured from the ratio of anti-Stokes/Stokes Raman modes corresponds more or less to a temperature at the sample surface with a size ˜1 µm^2^. Furthermore, it is expected that the central point of the laser beam impact would undergo the greatest heating effect, with a reduction at increasing displacement radii. Thus, the temperature is not uniform in the irradiated surface. The time for the laser to heat the monitored sample area (determined by the diameter of the slit entrance of the spectrometer) to a uniform temperature, must depend on the laser power. Therefore, this reveals the kinetic character of the probed phenomenon. Consequently, the calculated temperature is then an average temperature of probed surface and is different from the one obtained from DSC or XRD measurements where the sample is held under thermodynamic equilibrium.

## Conclusion

In this study, we have provided the Raman vibrational features of the orthorhombic phase of Cs_2_RuO_4_. By coupling group theory to quantum chemical analysis, we succeeded to attribute the Raman active modes of its orthorhombic room temperature structure. Raman laser heating caused multiple spectral changes and appearance of new modes characterized by an increase of the bandwidths of the bands. Thus, suggesting that a phase transition from an ordered to disordered symmetry has occurred. This latter was confirmed by high-temperature X-ray measurement. The new structural phase is connected with tilting and/or rotations of RuO_4_ tetrahedra, which leads to a disorder at the RuO_4_ sites. The temperature-phase transition was calculated from the anti-Stokes/Stokes ratio and compared with the one obtained from other measurements. Thus, the temperature-induced structural changes must play an important role in the adhesion of the Cs_2_RuO_4_ at the fuel's surface when undergoing thermal variations.

## Supporting Information

Supporting info item
