# Supplementary material for: Joint Raman spectroscopic and quantum chemical analysis of the vibrational features of Cs2RuO4
Source: J Raman Spectrosc. 2015 Apr 28;46(7):661–8. doi: 10.1002/jrs.4705 (PMC4608045; doi:10.1002/jrs.4705)
Supplement: Supplementary file 1 — Supporting info item [file jrs0046-0661-sd1.doc]

**Supporting information**

**Joint Raman spectroscopic and quantum chemical analysis of the vibrational features of Cs_2_RuO_4_**

### M. Naji,* F. Di Lemma, A. Kovács, O. Beneš, D. Manara, J-Y. Colle, G. Pagliosa, P. Raison, and R. J. M. Konings

*European Commission, Joint Research Centre (JRC), Institute for Transuranium Elements (ITU), Postfach 2340, 76125 Karlsruhe, Germany*

*E.mail :* [mohamed.naji@ec.europa.eu](mailto:mohamed.naji@ec.europa.eu)

**Experimental**

***Material synthesis***

**Table S1: Heat treatment sequence used for the synthesis of Cs2RuO4**

| Heat Treatment n°. | Temperature / K | Dwell Time /min | Crucible metal |
| --- | --- | --- | --- |
|  |  |  |  |
| 1 | 423 | 120 | Silver |
| 2 | 573 | 120 | Silver |
| 3 | 700 | 210 | Silver |
| *Sample cooled down slowly (3 K/ min) to room temperature.*  *Then, re-ground in agate mortar to improve homogeneity, compacted with a spatula and re-heated* | | | |
| 4 | 700 | 30 | Gold |
| 5 | 873 | 120 | Gold |
| Sample cooled down slowly (3 K/ min) to room temperature. XRD pattern was taken to judge the progress of the reaction.  Then, sample was re-ground to improve homogeneity, compacted with a spatula in silver/gold bot and re-heated | | | |
| 6 | 1075 | 180 | Gold |

**Instrumental Methods**

***X-ray diffraction***


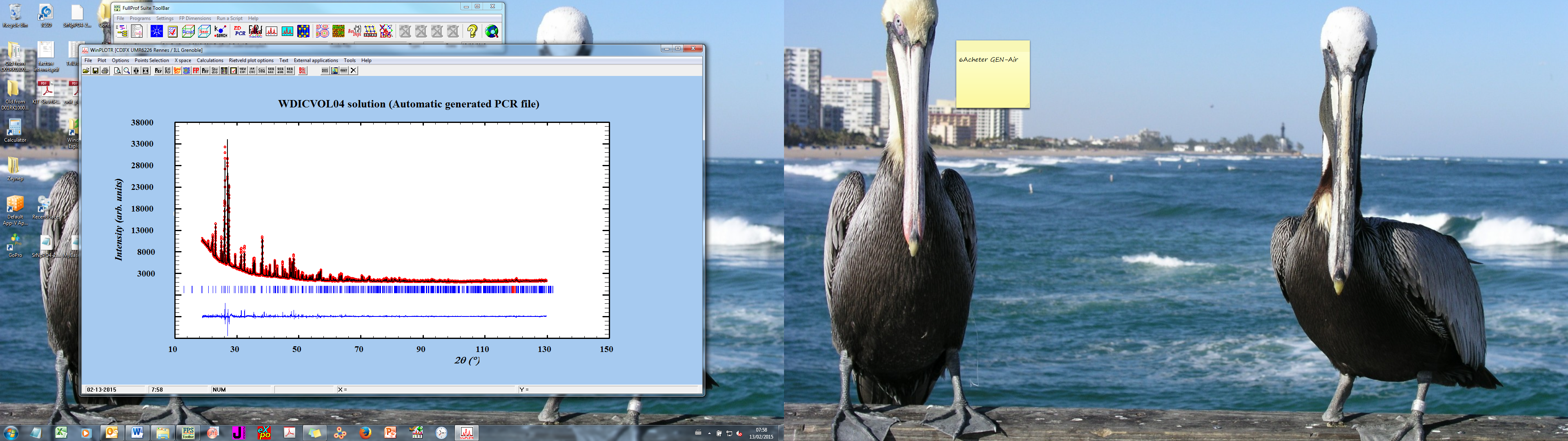


***Figure S1****:* *Comparison between the observed (Yobs, in red) and calculated (Ycalc, in black) X-ray diffraction pattern of Cs_2_RuO_4_ phase. Yobs-Ycalc, in blue is the difference between the experimental and calculated intensities. The Bragg reflections are marked in blue ticks. Measurement at λ = 1.54056Å.*

**Table S2. Atomic positions in the Cs_2_RuO_4_ compound**

| Atom |  | *Cs_2_RuO_4_* | | | | | | | |
| --- | --- | --- | --- | --- | --- | --- | --- | --- | --- |
|  |  |  | *This work* | | | *Fischer and Hope [1]* | | | |
|  | *Wyck.* | *x* | *y* | *z* | *B_iso_* | *x* | *y* | *z* | *B_iso_* |
| Ru | *4c* | 0.227(2) | 0.25 | 0.421(2) | 0.30 | 0.2266 | 0.25 | 0.4204 | 1.5 |
| Cs1 | *4c* | 0.6674 (2) | 0.25 | 0.4136(2) | 0.48 | 0.6665 | 0.25 | 0.4129 | 2.2 |
| Cs2 | *4c* | 0.4897 (3) | 0.25 | 0.7958(3) | 0.48 | 0.4900 | 0.25 | 0.7957 | 1.9 |
| O1 | *4c* | 0.3033(4) | 0.25 | 0.5998(4) | 0.5 | 0.2990 | 0.25 | 0.5656 | 2.5 |
| O2 | *4c* | 0.0158(3) | 0.25 | 0.4144(4) | 3.7 | 0.0208 | 0.25 | 0.4163 | 3.5 |
| O3 | *8d* | 0.3002(3) | 0.5045(4) | 0.3725(4) | 4.2 | 0.3010 | 0.4720 | 0.3508 | 3.1 |

[1] Fischer, D.; Hoppe, R, Zeitschrift fuer Anorganische und Allgemeine Chemie (1950) (DE) (1990) 591, p87-p94
